# Yak ( *Bos grunniens* ) Meat Peptides: Effects on Immunity, Hypoxia Tolerance, and Antioxidant Capacity in Hypotonic Hypoxia Mice

**DOI:** 10.1002/fsn3.70522

**Published:** 2025-06-27

**Authors:** Gaiming Zhao, Ying Zhang, Ke Wang, Long Xu, Mancai Yin, Xiangying Kong, Chaozhi Zhu

**Affiliations:** ^1^ Henan Key Lab of Meat Processing and Quality Safety Control Henan Agricultural University Zhengzhou China; ^2^ College of Food Science and Technology Henan Agricultural University Zhengzhou China; ^3^ National Beef Cattle and Yak Industry Technology System Qinghai Yak Breeding and Promotion Service Center Xining China; ^4^ National Beef Cattle and Yak Industry Technology System Qinghai Haibei Animal Husbandry and Veterinary Science Research Institute Haibei China

**Keywords:** antioxidant activity, hypotonic hypoxia model, in vitro digestion, yak meat peptides

## Abstract

The present study focused on the investigation of yak ( *Bos grunniens* ) meat peptides on immunity, antioxidant capacity, and hypoxia tolerance in hypotonic hypoxic mice using malondialdehyde (MDA), superoxide dismutase (SOD) kit, and so on. Yak protein powder was extracted first, and then peptides were obtained by INFOGEST static simulation of human gastrointestinal tract digestion. The results demonstrated that yak meat peptides increased the content of immune cells, oxygen transport capacity, and antioxidant enzyme activities in mice, while reducing oxidative stress damage in the body. In addition, protein expression of hypoxia‐related genes hypoxia‐inducible factor (HIF) and erythropoietin (EPO) was improved. This study can provide a theoretical foundation for reducing the harm of reactive oxygen species and free radicals to the human body and for the development and utilization of yak bioactive substances.

## Graphical abstract

Yak meat peptides can increase immune cell content, oxygen transport capacity, and antioxidant enzyme activities in hypotonic hypoxic mice. Meanwhile, they can reduce oxidative stress, thus enhancing the immunity, antioxidant capacity, and hypoxia tolerance of these mice. They also upregulate hypoxia‐related genes HIF and EPO. This study provides a theoretical basis for mitigating oxidative damage and advancing the development of yak‐derived bioactive substances.

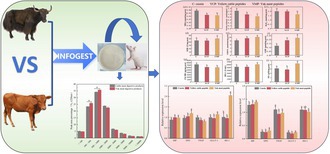

## 1 Introduction

Yak ( *Bos grunniens* ) is a kind of cattle born on the Tibetan Plateau, living all year round in a plateau environment with an average temperature below 0°C, low pressure, and lack of oxygen and strong ultraviolet rays (Niu et al. 2004). Yellow cattle are an important part of beef consumption in the Central Plains, especially dominating the local specialty diet and high‐end beef market. However, Highland yak ( *B. grunniens* ) is richer in proteins and amino acids than yellow cattle (*Simmental*), and it exhibits superior adaptability to the plateau environment. Specifically, yaks all live in high altitude areas, but they have excellent physiological adaptations, which enable them to better cope with the hypoxic conditions at high altitudes (Ayalew et al. 2021; Luo et al. 2006). Tibetan nomads live in the same oxygen‐deficient environment and have an extremely low intake of vegetables, fruits, and grains (Guo et al. 2014). Yak meat, as the main source of protein, accounts for a very high proportion of the dietary structure of the local Tibetan population (Rhode et al. 2007; Zhou et al. 2021). Proteins in food not only serve as nutrients, but also regulate the physiological functions of the human body (Chakrabarti et al. 2018), so the intake of yak and its products has an important impact on the adaptation of Tibetans to the low‐pressure hypoxic environment of the plateau. Yak meat reacts with digestive enzymes in the human digestive process to generate a large number of active peptides (Arihara et al. 2021). Small peptides are absorbed after binding to transporter proteins. High molecular weight peptides require enzymatic hydrolysis to further break them down into small peptides and amino acids before they can be absorbed (Qiao et al. 2025; Wang et al. 2024). These active peptides play an important role in human health and disease prevention, as evidenced by their antioxidant, anti‐inflammatory, immunomodulatory, and metabolism‐regulating properties (Bhandari et al. 2020).

Antioxidant peptides, owing to their potential in scavenging reactive oxygen species (ROS) and alleviating diseases linked with oxidative stress, have become a significant subject of research (Chen et al. 2021; Meng et al. 2024). In biological context, antioxidant peptides are categorized into endogenous and exogenous active peptides. Endogenous active peptides are naturally present in cells, such as glutathione and carnosine (Wong et al. 2020), whereas exogenous antioxidant peptides are produced in vitro through gastrointestinal digestion of dietary foods. Current research on antioxidant peptides indicates that small molecule peptides (< 1 kDa), exhibit enhanced bioactivity, including 1,1‐diphenyl‐2‐picrylhydrazyl (DPPH) radical scavenging and Fe^2+^ chelating properties (Xia et al. 2012; Mundi and Aluko 2014; Wang et al. 2021). Building on these findings, our study focuses on exploring the antioxidant potential of yak meat‐derived peptides.

ROS are unavoidable by‐products of cellular respiration. Hypoxia reduces aerobic oxidative respiration and decreases the rate of electron transfer in the mitochondria, resulting in an increase in ROS content, causing oxidation stress (OS) and irreversible damage to the organism (Chen et al. 2018). The human body will be affected by the increase of ROS when it is exposed to external stimuli, such as hypoxia, ultraviolet light, radiation, air pollution, and so on. Excessive ROS can cause OS in the body, damaging cell membranes, oxidizing proteins, and damaging the body's DNA and RNA (Rahman et al. 2012). OS damages DNA and RNA in the brain, which can lead to protein oxidation. The products of lipid peroxidation can cross link with amino acids to cause toxicity, which ultimately leads to neurological lesions or neuronal cell death, inducing diseases such as Alzheimer's disease (AD), Parkinson's (PD), and amyotrophic lateral sclerosis (ALS) (Teleanu et al. 2022). The Qinghai‐Tibet Plateau (average elevation > 4000 m) exacerbates the production of oxidative stress, and plateau herders are more susceptible to the adverse effects of oxidative stress (Demirci‐Çekiç et al. 2022; Beall et al. 2010). Consequently, it is urgent to reduce the harmful effects of ROS and free radicals on the human body during hypoxia.

By comparing the structural characteristics and functional performance of yak meat peptides with those of yellow cattle peptides, we systematically investigated the superior antioxidant capacity and hypoxia tolerance of yak‐derived peptides. Moreover, the molecular mechanism by which yak meat peptides improve hypoxia tolerance was revealed. These findings demonstrate significant potential in mitigating the harmful effects of ROS and free radicals, laying a theoretical foundation for future development and utilization of bioactive peptides from yak meat in human health applications.

## 2 Materials and Methods

### 2.1 Materials and Reagents

Malondialdehyde (MDA), superoxide dismutase (SOD), glutathione peroxidase (GSH‐Px), total antioxidant capacity (T‐AOC), and reduced glutathione (GSH) test kits were purchased from Suzhou Grace Biotechnology Co. Ltd. (Suzhou, China); hydrogen peroxide (H_2_O_2_), protein carbonyl content, and creatine kinase isoenzyme MB (CK‐MB) test kits were purchased from Wuhan Elite Bioscience and Technology Co. Ltd. (Wuhan, China); nitric oxide (NO) and heme oxygenase (HO‐1) test kits were purchased from Biyuntian Biologicals Co. (Shanghai, China). Other reagents used were analytical chemicals, which were purchased from China Sinopharm Group Chemical Reagents Co. Ltd. (Shanghai, China).

### 2.2 Preparation of Yak Meat Peptides and Yellow Cattle Peptides

#### 2.2.1 Preparation of Beef Protein Powder

Yak meat has the capacity to enhance the immunity, antioxidant ability, and hypoxia tolerance of animals' bodies. Previous laboratory studies revealed that this is attributed to protein rather than fat. Yak and yellow cattle knuckle (*Musculus quadriceps femoris*) were both selected from 24‐month‐old, 300‐kg carcass (males). Yak meat was selected from naturally free‐range yaks in Shigatse, Tibet, which is at an altitude of 4000 m. Yellow cattle meat was selected from Simmental cattle from Yisai Beef Cattle Slaughterhouse (Henan, China). The samples were frozen at −80°C after collection.

After thawing, we removed the surface fat and fascia from the cattle chuck tender (*M. supraspinatus*). Then, mince it using a meat grinder equipped with a 6 mm hole plate. The meat pellets were evenly spread in a cooking bag (thickness not more than 2 cm) and placed in a 72°C electric thermostatic water bath (HHS‐21‐8 Shanghai Boxun Co. Ltd. Shanghai, China) and heated in the water bath until the center temperature of the meat pellets reached 70°C. Dry the water on the surface of the meat pellets, and then place the meat pellets in the refrigerator at −40°C for pre‐freezing after the temperature of the meat pellets was restored to room temperature. The frozen meat particles were put into a vacuum freeze dryer (Scientz‐30ND, Ningbo Xinzhi Biotechnology Co. Ltd., Ningbo, China) for freeze drying to remove most of the water, and then grounded after freeze drying to obtain ground beef. Forty grams of ground beef was added to 15 times the volume of a mixture of dichloromethane and methanol (2:1) for extraction and degreasing. The degreased meat powder was spread flat in a tray and placed in a ventilated kitchen to evaporate the organic reagents. The beef protein powder was collected through a 20‐mesh sieve.

#### 2.2.2 Simulates In Vitro Digestion

Gastrointestinal food digestion was simulated in vitro using INFOGEST static in reference to André Brodkorb et al. (2019). Beef protein powder was added to simulated saliva with a mass ratio of 1:1. Perform homogenization at 9600 rpm and 13,400 rpm respectively for 30 s twice, with a 30‐s interval between the two homogenization times, to simulate oral digestion. An equal volume of simulated gastric fluid was added to the homogenized sample solution and mixed well. The pH was adjusted to 3.0 with 5 mol/L HCl then added to the already configured pepsin solution. Finally the pepsin activity in these mixed solutions was made to reach 2000 U/mL. The digestion was incubated at 37°C for 2 h with shaking to simulate the gastric phase environment. Immediately after the incubation, 5 mol/L NaOH was added to inactivate pepsin, while the pH of the digestive solution was adjusted to 7.0. Finally, equal volumes of simulated intestinal fluid and trypsin premix were added to bring the final enzyme activity to 100 U/mL, and the incubation was continued at 37°C for 2 h. The digestive enzymes in the final simulated in vitro digested product were inactivated by heating the mixture in boiling water for 5 min. The configuration of simulated saliva, gastric, and intestinal fluids required for digestion is shown in Table S1.

#### 2.2.3 Peptide Preparation

The in vitro simulated digestive solution was mixed with anhydrous ethanol (three times the volume of the digestive solution) by shaking and left to stand for 30 min. After centrifugation (11,000 rpm × 15 min) at 4°C (Beckman Allegra 64R, Beckman Coulter, Brea, California, American), the supernatant was concentrated (HHS‐21‐8, Shanghai Boxun Co. Shanghai, China). Then, the concentrated liquid was freeze dried (Scientz‐30nd, Ningbo Xinzhi Biotechnology Co., Ningbo, China). In the same way, yellow cattle peptide was produced.

#### 2.2.4 Determination of Molecular Weight Distribution of Peptides

The molecular weight distribution of the peptides after digestion of yak meat and cattle meat was determined using a Waters 2695 high performance liquid chromatograph. The conditions of HPLC were as follows: column, TSK gel2000 SWXL 300 mm × 7.8 mm gel column, mobile phase was acetonitrile:water:trifluoroacetic acid 40:60:0.1 (volume ratio), the detection wavelength was 220 nm, the flow rate was 0.5 mL/min, the column temperature was 30°C. Sample preparation: The sample was taken in a 10 mL volumetric flask, diluted to scale with mobile phase, sonicated for 5 min, centrifuged, and filtered through a microporous membrane for injection. The standard substances used to establish the standard curve and their corresponding relative molecular weights were cytochrome C (MW12384), aprotinin (Mw6500), bacitracin (Mw1422), ethanine‐acetate‐tyrosine‐arginine (Mw451), and ethanedioic acid–ethanedioic acid–ethanedioic acid (Mw189), respectively.

### 2.3 Measuring Antioxidant Activity In Vivo

#### 2.3.1 Animals

Seventy‐five specific pathogen‐free (SPF) grade c57 male mice (6–8 weeks old, 20 ± 2 g) were purchased from Shanghai Slaughter Laboratory Animal Co. In the SPF animal room, mice were housed in poly‐sulfone (PSU) plastic individually ventilated cages (G4 IVC system) measuring 370 mm in length, 157 mm in width, and 180 mm in height. Following that, they were housed in cages under typical laboratory circumstances. The temperature was within the range of 20°C–26°C, humidity was between 40% and 70%, and there was a 12‐h light/dark cycle. Food was supplied regularly, and access to water was unrestricted. All animal experiments complied with ARRIVE (Animal Research Reporting of In Vivo Experiments) guidelines.

#### 2.3.2 Experimental Design

The mice were allowed to adapt to the environment under consistent conditions for a period of 1 week. These conditions included a temperature of 22°C ± 2°C, relative humidity of 40%–70%, a 12‐h light/dark cycle, and free access to standard laboratory chow and drinking water. They were randomly divided into three groups (casein group, yellow cattle peptide group, and yak meat peptide group) with 25 animals in each group. The blank control group was the casein group. The mice were fed normal casein chow and gavaged daily with saline according to the gavage volume of the experimental group. The experimental groups were divided into the yellow cattle peptide group and the yak meat peptide group. The mice were fed a low casein diet (10.125 g of protein per kilogram of feed) and were gavaged with yellow cattle peptide and yak meat peptide at a daily gavage dose of 1500 mg/kg, respectively.

#### 2.3.3 Determination of Hypoxia Tolerance in Mice

After 30 days of feeding according to the above experimental method, mice were subjected to a normoxia tolerance test, sodium nitrite poisoning survival test, and acute cerebral ischemic hypoxia test. If any two of the three tests are positive, it can be determined that the test sample has the effect of hypoxia resistance.

##### 2.3.3.1 Normoxia Tolerance Test

One hour after the last gavage, the mice in each group were placed into 250 mL ground bottles, each containing 5 g of sodium lime (one mouse per bottle). The bottles were sealed with petroleum jelly, capped tightly to make them airtight. Then timing was started, and the time of death of the mice due to hypoxia was recorded, with respiratory arrest as an indicator.

##### 2.3.3.2 Sodium Nitrite Poisoning Survival Test

One hour after the last gavage, mice in each group were injected intraperitoneally with sodium nitrite (0.1 mL/10 g) and immediately timed to record the survival time of the mice.

##### 2.3.3.3 Acute Cerebral Ischemic Hypoxia Test

One hour after the last gavage, mice in each group were severed one by one from the neck. The time from the severing of the head to the cessation of open‐mouth panting of the mice was recorded immediately by a stopwatch.

#### 2.3.4 Determination of Blood Physiological Indexes and In Vivo Antioxidant Activity Indexes in Mice

The XN‐1000 V hematology analyzer (HysonMedicom Medical Electronics, Shanghai, China) was used for mouse blood analysis. After feeding mice for 30 days, orbital venous blood was taken 1 h after the last gavage. MDA and T‐AOC content, SOD and GSH‐Px activities were determined in strict accordance with the kit instructions.

### 2.4 Establishment of Hypotonic Hypoxia Mouse Model

In 2.3.3.1 normobaric hypoxia test, the normobaric hypoxia model was successfully established when the mice stopped breathing. Immediately dissect the mice, take out the myocardial tissue, and put it into liquid nitrogen for quick freezing. Subsequently, the myocardial tissue was frozen at −80°C for subsequent experiments. The content of MDA, H_2_O_2_, protein carbonyl, GSH, CK‐MB, NO, and the activity of SOD, GSH‐Px, and HO‐1 were measured in mouse myocardial tissue cells in strict accordance with the kit instructions.

### 2.5 Determination of Hypoxia‐Related Gene Expression in Myocardial Tissues of Hypoxic Mice(qRT‐PCR)

The myocardial tissue was placed into a centrifuge tube. Total RNA was extracted by means of Trizol reagent (California, USA) following the manufacturer's instructions. Subsequently, the extracted RNA was reverse transcribed to obtain cDNA with the help of Enzymes Mix and 5× RT Super Mix Buffer. A total volume of 20 μL of cDNA was used as the template to configure the qPCR reaction system. To this, 10 μL of 2× M5 HiPer SYBR Premix Es Taq, 1 μL of forward primer, 1 μL of reverse primer, 1 μL of cDNA, and 7 μL of ddH_2_O were added, respectively. The reaction was carried out in a Roche fluorescence quantitative PCR plate and then put into a fluorescence quantitative PCR instrument. The PCR cycling parameters used were: initial denaturation at 95°C for 10 min, followed by 95°C for 5 s and 57°C for 30 s, then cycled 40 times. The dissolution curve was as follows: 95°C for 15 s and then 60°C for 1 min. β‐Actin was used as the internal reference gene, and three replicate wells were made for each sample. The 2^−ΔΔCT^ method was used to calculate the relative mRNA levels of target genes. The primers are shown in Table S2.

### 2.6 Determination of Hypoxia‐Related Protein Expression in Myocardial Tissues of Hypoxic Mice (Western Blot)

The myocardial tissue samples were lysed on ice with 100 μL of radio immunoprecipitation assay (RIPA) buffer (containing phenyl methyl sulfonyl fluoride (PMSF) and phosphatase inhibitors) and cryogenically ground. The samples were lysed on ice for 10 min and shaken 3–5 times. Then, centrifuge at 4°C (12,000 × g for 10 min) and transfer the supernatant to another centrifuge tube. Take a small amount of protein for bicinchoninic acid (BCA) protein concentration assay (100‐fold dilution). After configuring the polyacrylamide gel, the parameters were set as follows: concentration gel 80 V, 30 min, separation gel 120 V, 1.5 h. After electrophoresis was aborted, membrane transfer was carried out. Polyvinylidene fluoride (PVDF) membranes were activated in methanol for 15 min and flattened on a membrane transfer device for 35 min. The membrane was rapidly placed in the sealing solution for 15 min at 4°C, rinsed three times with Tris‐buffered saline Tween (TBST) and placed in primary antibody at 4°C overnight. On the next day, the membrane was rinsed three times with TBST (10 min/time) and incubated in secondary antibody at room temperature for 2 h. After rinsing three times with TBST (10 min/time), the PVDF membrane was exposed to enhanced chemiluminescence (ECL) luminescent solution for developing. The exposure images were scanned using ImageJ software for grayscale analysis and the results were analyzed.

### 2.7 Statistical Analysis

Each experiment was repeated on three replicates, and the results are presented as mean values accompanied by standard deviation. Statistical analysis was performed using SPSS 26.0 (SPSS Inc., Chicago, USA), employing analysis of variance (ANOVA) at a significance level of *p* < 0.05. The graphs in this study were plotted using Origin 2024 software (OriginLab Corp., Northampton, MA, USA).

## 3 Results and Analysis

### 3.1 The Difference in Molecular Weight Distribution of Peptides After Digestion of Yak and Cattle Protein Powders

**FIGURE 1:**
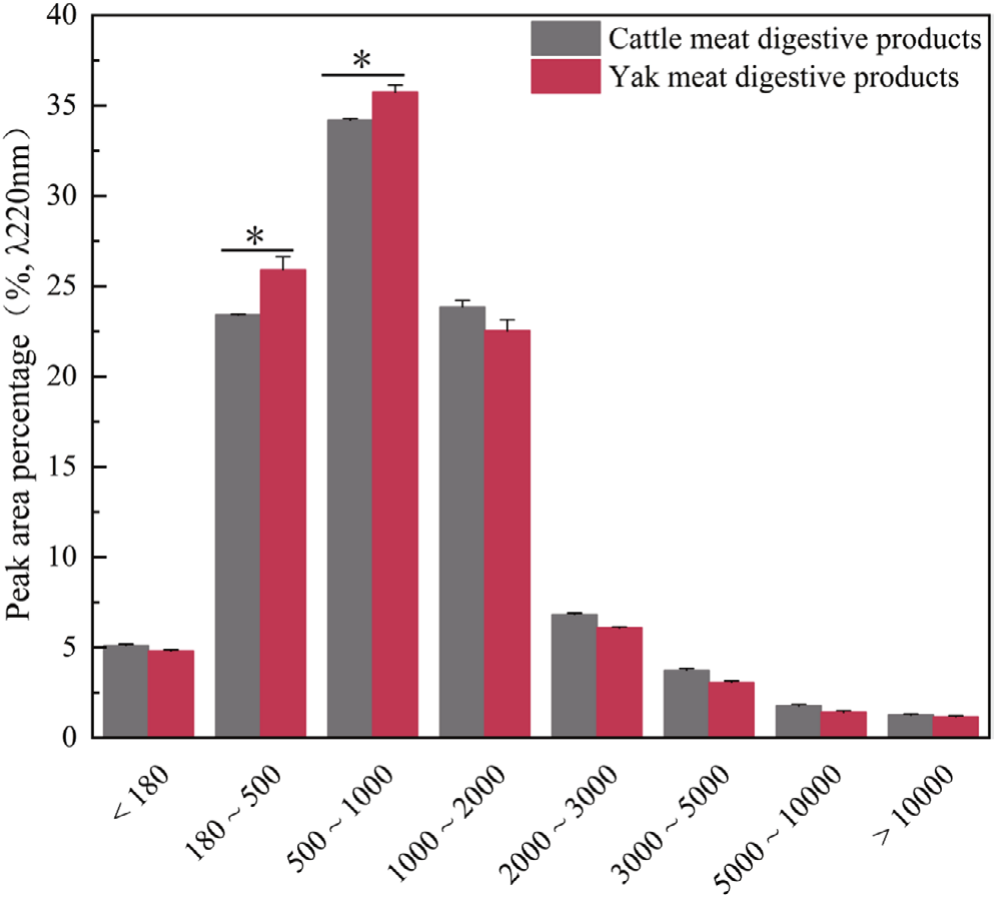
Molecular weight distribution of polypeptides after in vitro simulated digestion of yak and yellow cattle protein extract. The unit of the horizontal coordinate is Da. * Indicates differences between samples (*p* < 0.05).

Once proteins are digested in simulated in vitro environments, the biological activity of the resulting hydrolysates and the potency of peptides largely rely on the molecular weight distribution and also the compositional and sequential layout of amino acids (Hong et al. 2019). As shown in Figure 1, the peptide content in the simulated human in vitro digestive products of yak protein powder was significantly higher than that of yellow cattle in the molecular weight ranges of 180–500 and 500–1000 Da (*p* < 0.05). However, the molecular weights of antioxidant peptides from meat proteins and their by‐products that have been identified in current research are usually in the range of 180–1000 Da. For example, Ohata et al. (2016) isolated and identified antioxidant peptides with a molecular weight of 406.26 Da in a meat sauce made from pork; the molecular weights of antioxidant peptides isolated and purified from Jinhua ham and Xuanwei ham are 564.4 and 505.2 Da, respectively (Zhu et al. 2013; Xing et al. 2016). Numerous studies have demonstrated that the molecular weight distribution of antioxidant peptides generally falls within the range of 180–1000 Da. From this, it is inferred that the peptides from yak meat protein powder simulated with in vitro digestion may have antioxidant properties.

### 3.2 Effects of Yak Meat Peptides on Hypoxia Tolerance in Mice

As depicted in the Table 1, the survival time of mice gavaged with yellow cattle peptide and yak meat peptide was significantly greater than that of mice in the casein group in the normoxia tolerance experiment (*p* < 0.05). In the nitrite poisoning experiment, both yak meat peptide and yellow cattle peptide significantly increased the survival time of mice (*p* < 0.05), and the effect of yak meat peptide was better than that of yellow cattle peptide. Therefore, yak meat peptide has stronger protection ability and oxygen transportation ability for hemoglobin. According to the statistical method and result determination standard of the hypoxia resistance test method in the “Health Food Functional Performance Test and Evaluation Methods (2023 Edition),” if any two of the three experiments are positive, it can be determined that the test sample has the effect of hypoxia resistance. Therefore, both yak meat peptide and yellow cattle peptide have the effect of hypoxia tolerance compared with casein.

**TABLE 1:** Effect of yak meat peptides on hypoxia tolerance in mice.

Groups	Standing normal pressure and hypoxia survival time (min)	Exposed to nitrite poisoning survival time (min)	Acute cerebral ischemia survival time (min)
Casein	37.98 ± 2.12^b^	16.07 ± 1.52^b^	27.07 ± 2.05^a^
Cattle meat peptides	44.84 ± 3.85^a^	20.82 ± 3.24^a^	24.27 ± 3.52^a^
Yak meat peptides	43.32 ± 3.69^a^	24.25 ± 3.86^a^	19.30 ± 1.02^b^

*Note:* For each column, different lowercase letters in the table indicate significant differences between samples (*p* < 0.05).

### 3.3 Effects of Yak Meat Peptide on Blood Physiological Indexes and In Vivo Antioxidant Activity Indexes in Mice

Yak meat peptides significantly increased the percentage of neutrophils, mean hemoglobin content, and concentration in the blood of mice compared to yellow cattle peptides (Table 2). Neutrophils, innate immune cells, are immune cells accounting for a relatively large proportion in the blood and form the first line of defense against pathogens (Blanter et al. 2021). Hemoglobin has a high oxygen‐carrying capacity and will lose its ability to transport oxygen once it escapes into the plasma (Ahmed et al. 2020). People living in high‐altitude areas often have a relatively high hemoglobin content. This is because the body produces more hemoglobin to carry enough oxygen in order to adapt to the environment with thin oxygen (Siebenmann et al. 2024). Therefore, the intake of yak meat peptides significantly increased the hemoglobin concentration as well as the neutrophil content in the blood of the mice, which led to an increase in immunity and the ability of red blood cells to transport oxygen.

**TABLE 2:** Effects of yak meat peptides on blood physiological indicators in mice.

Indicator	Casein	Cattle meat peptides	Yak meat peptides
Neutrophil percentage (%)	7.60 ± 1.08^b^	9.67 ± 0.37^b^	14.87 ± 6.40^a^
Basophils percentage (%)	0.10 ± 0.00^a^	0.03 ± 0.05^b^	0.03 ± 0.05^b^
Absolute basophils (10^9^/L)	0.01 ± 0.00^a^	0.00 ± 0.01^b^	0.00 ± 0.01^b^
Mean hemoglobin content (pg)	13.87 ± 0.05^b^	13.90 ± 0.09^b^	14.20 ± 0.18^a^
Mean hemoglobin concentration (g/L)	293.33 ± 2.25^b^	291.00 ± 5.59^b^	303.00 ± 2.68^a^
Nucleated red blood cell count (10^9^/L)	0.02 ± 0.02^b^	0.06 ± 0.03^a^	0.04 ± 0.01^b^
Nucleated red blood cell (%)	0.13 ± 0.14^c^	0.67 ± 0.23^a^	0.37 ± 0.05^b^

*Note:* For each line, different lowercase letters in the table indicate significant differences between samples (*p* < 0.05).

**FIGURE 2:**
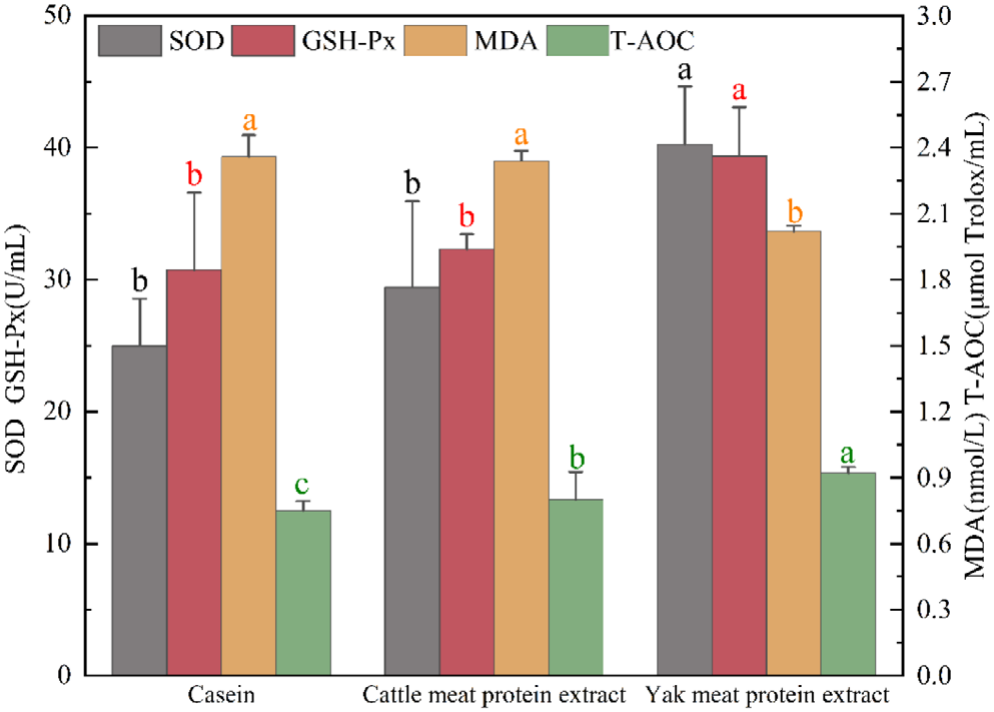
The effects of yak meat protein extract on SOD, GSH‐Px activity, and MDA, T‐AOC content in mouse serum. Different lowercase letters of the same color represent significant differences between samples (*p* < 0.05).

SOD can reduce the damage of superoxide anion to cells and play a key role in antioxidant. When cells are subjected to oxidative stress they accumulate many free radicals and ROS, and these peroxides lead to some degree of oxidative damage (Stephenie et al. 2020). GSH‐Px, in conjunction with SOD and catalase, serves as an antioxidant enzyme and they together constitute an enzymatic antioxidant system which is able to lessen the oxidative cellular harm brought about by ROS and free radicals. GSH‐Px reduces peroxide levels in the body by catalyzing GSH, which also acts as an erythrocyte enzyme to protect hemoglobin from oxidative stress (Pei et al. 2023). According to the Figure 2, the activity of SOD and GSH‐Px in the serum of mice in the yak meat peptide group was significantly higher than that of mice in the casein and yellow cattle peptide groups (*p* < 0.05). The above results indicated that yak meat peptide could significantly increase the activity of SOD and GSH‐Px compared with yellow cattle peptide and casein, improve the antioxidant capacity of the organism, and reduce the oxidative stress damage caused by reactive oxygen species and free radicals.

MDA is one of the end products of intracellular peroxidation of polyunsaturated fatty acids. When the body experiences oxidative stress injury, the increase in ROS leads to elevated levels of MDA. This is why MDA is also regareded as a biomarker of lipid peroxidation and OS (Del Rio et al. 2005). As presented in the Figure 2, the MDA content in the casein and yellow cattle peptide groups was significantly higher than that in the yak meat peptide group (*p* < 0.05). This suggested that antioxidant enzymes and antioxidants in the body scavenge reactive oxygen species as well as free radicals well and inhibit lipid peroxidation, resulting in less oxidative damage to tissue cells.

T‐AOC is an index reflecting the comprehensive antioxidant capacity of the body's antioxidant enzymes and all antioxidant substances (He et al. 2024). There were significant differences in the T‐AOC contents of mice among the three groups (*p* < 0.05). The group with yak meat peptides had the highest T‐AOC content, which was notably higher than that of the yellow cattle peptide group (Figure 2). The results suggested that yak meat peptide, compared with yellow cattle peptide and casein, could significantly increase the total antioxidant capacity in serum of mice, improve the protection of the organism during oxidative stress, and reduce the oxidative stress damage of cells caused by ROS and free radicals.

### 3.4 Effects of Yak Meat Peptides on Myocardial Tissue in Hypotonic Hypoxic Mice

**FIGURE 3:**
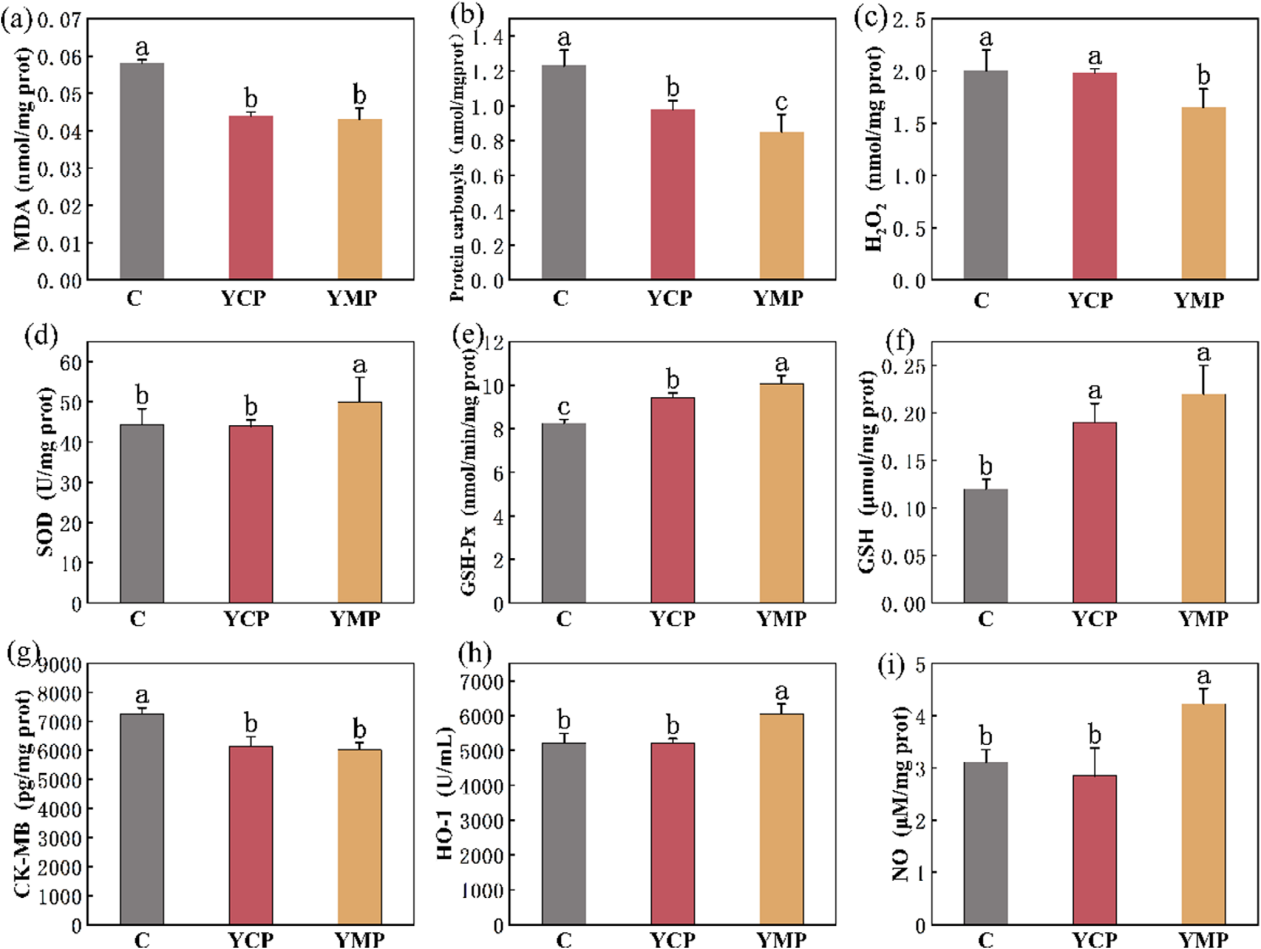
The effects of yak meat peptides on myocardial tissue in hypotonic hypoxic mice. C, YCP, and YMP represent casein, yellow cattle peptides, and yak meat peptides, respectively. Contents of malondialdehyde (MDA) in mouse myocardial tissue (a). Contents of hydrogen peroxide (H_2_O_2_) in mouse myocardial tissue (b). Contents of protein carbonyls in mouse myocardial tissue (c). Activity of superoxide dismutase (SOD) in mouse myocardial tissue (d). Activity of glutathione peroxidase (GSH‐Px) in mouse myocardial tissue (e). Contents of glutathione (GSH) in mouse myocardial tissue (f). Contents of creatine kinase isoenzyme (CK‐MB) in mouse myocardial tissue (g). Activity of heme oxygenase (HO‐1) in mouse myocardial tissue (h). Contents of nitric oxide (NO) in mouse myocardial tissue (i). Different lowercase letters for each index indicate the differences among samples (*p* < 0.05).

MDA content can accurately reflect the degree of lipid oxidation and the degree of cellular oxidative stress. The higher the MDA content, the higher the degree of cellular oxidative stress and the higher the oxidative cellular damage caused (Zhan et al. 2023). As revealed by Figure 3a, although there was no significant difference in the content of MDA in the myocardial tissue cells of mice in the yak meat peptide group and yellow cattle peptide group, it was significantly lower than that in the casein group (*p* < 0.05). This implied that yak meat peptide and yellow cattle peptide may have helped the body to reduce the attack of free radicals on lipids. In a hypotonic and hypoxic environment, the antioxidant property and the ability to inhibit lipid peroxidation of casein were significantly inferior to those of yellow cattle peptide and yak meat peptide. Proteins are the most direct carriers of oxidative damage to cells, and protein carbonyls serve as biomarkers of the level of oxidative stress in the body (Akagawa 2021). Similar to the change in MDA content, the content of protein carbonyls also reflects the antioxidant effect of yak meat peptides. Shown in Figure 3b, there was a significant difference (*p* < 0.05) in the protein carbonyl content in the myocardial tissues of mice in all three groups. The lowest protein carbonyl content was found in the yak meat peptide group. This revealed that proteins in mice myocardial tissues in the yak meat peptide group were less attacked by free radicals in hypotonic hypoxic environment. Alternatively, they were scavenged by antioxidant enzymes in the body before the free radicals caused oxidative damage to the proteins, thus maintaining the structural and functional integrity of the proteins and keeping the protein carbonyl content at a low level. H_2_O_2_ is a by‐product produced during the metabolism of ROS. Elevated H_2_O_2_ content indicated that ROS production had increased, oxidative stress in the body had been intensified, and lipid peroxidation damage had been increased. The content of H_2_O_2_ in the myocardial tissues of mice in the yak meat peptide group was significantly lower than that in the casein and yellow cattle peptide groups (*p* < 0.05; Figure 3c). The above results demonstrated that yak meat peptide could significantly reduce the content of H_2_O_2_ in mouse myocardial tissues in a hypotonic hypoxic environment and reduce the OS damage caused by H_2_O_2_ to mouse myocardial tissue cells.

SOD can promote the disproportionation reaction of superoxide to generate hydrogen peroxide and oxygen molecules, thus scavenging free radicals, preventing the chain reaction of free radicals, and exerting a protective effect on the organism. Results of Figure 3d exhibited that the highest SOD activity was found in the myocardial tissues of mice in the yak meat peptide group, which was significantly greater than that in the casein and yellow cattle peptide groups (*p* < 0.05). This indicated that in the hypotonic hypoxic environment, yak meat peptides could reduce the damage of ROS and free radicals on the oxidative stress of mouse myocardial tissue cells, and improve the antioxidant capacity of mice. GSH‐Px converts GSH to oxidized glutathione (GSSG), which reduces hydrogen peroxide or organic hydroperoxides to water or the corresponding alcohols in order to prevent cells from oxidative damage (Wang et al. 2023). From the results of Figure 3e, there was a significant difference in the activity of GSH‐Px in the myocardial tissues of mice in all three groups, with the highest activity of GSH‐Px in the yak meat peptide group (*p* < 0.05). GSH in food does not directly increase the glutathione content in the body, but it will stimulate the production of endogenous glutathione in the cells and improve the antioxidant capacity of the body (Al‐Temimi et al. 2023). GSH directly scavenges reactive oxygen and reactive nitrogen radicals in the body on the one hand, and on the other hand participates in the scavenging reaction of hydrogen peroxide and organic hydroperoxides as a substrate for GSH‐Px. As seen in Figure 3f, the GSH content in the myocardial tissue cells in the yak meat peptide group was the highest and significantly higher than that in the casein group (*p* < 0.05). The high content of GSH provides sufficient substrate for GSH‐Px to ensure the efficient operation of the body's antioxidant defense system.

CK‐MB is one of the sensitive markers available for assessing myocardial injury (Razavi et al. 2013). Figure 3g showed that CK‐MB activity in myocardial tissues of mice in the yak meat peptide group was the lowest, which was significantly lower than that in the casein group (*p* < 0.05). The above results indicated that yak meat peptide and yellow cattle peptide could reduce the OS damage in myocardial tissue of mice compared with casein. In hypotonic hypoxia environments, they had a certain protective effect on myocardial tissue. HO‐1 breaks down heme into unstable iron, biliverdin, and nitric oxide, where biliverdin is further reduced to the antioxidant bilirubin by biliverdin reductase. Heme degradation facilitates the reduction of pro‐oxidant effect of heme and protects the cells. According to Figure 3h, the content of HO‐1 in myocardial tissues of mice in the yak meat peptide group was the highest and significantly higher than that of casein and yellow cattle peptide (*p* < 0.05). Therefore, under hypotonic hypoxia, yak meat peptide significantly increased the activity of HO‐1, reduced cell damage by oxidative stress, and improved the antioxidant capacity of mice. NO, as a major signal and effector molecule mediating the body's response to hypoxia, can improve blood flow and oxygen supply through vasodilation, while regulating energy metabolism to reduce oxygen consumption (Umbrello et al. 2013). Illustrated in Figure 3i, the highest NO content in myocardial tissues of mice in the yak meat peptide group was significantly higher than that in the casein and yellow cattle peptide groups (*p* < 0.05). The results implied that under hypotonic hypoxia, yak meat peptide could improve the oxygen supply of hypoxic mice through vasodilation, and at the same time reduce the oxygen consumption of body metabolism. Clearly, taking yak meat peptides decreased the damage of reactive oxygen species to the cardiovascular system and raised the antioxidant capacity of mice.

### 3.5 Effect of Yak Peptide on mRNA Expression of HIF, EPO, VEGFA, GLUT1, and HO‐1 in Mouse Myocardial Tissue

**FIGURE 4:**
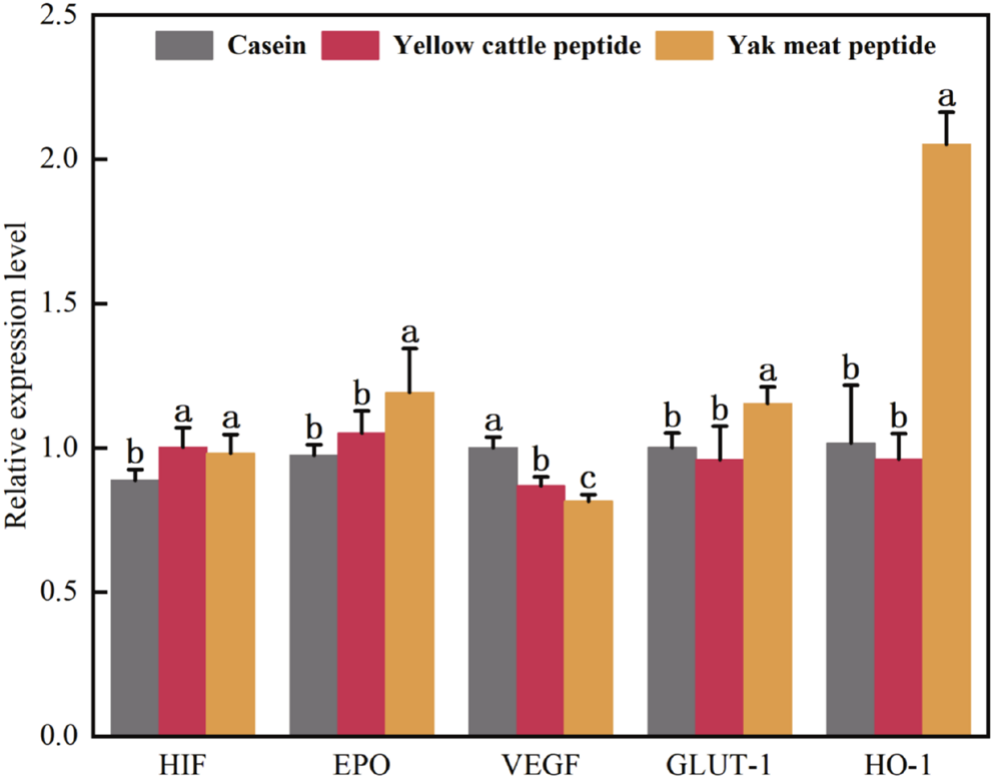
The effects of yak meat peptides on hypoxia‐related gene expression in mouse myocardial tissue. HIF, EPO, VEGFA, GLUT‐1, and HO‐1 represent hypoxia‐inducible factors, erythropoietin, vascular epidermal growth factor, glucose transporter protein, and heme oxygenase in mouse myocardial tissues, respectively. Different lowercase letters for each gene indicate the differences among samples (*p* < 0.05).

Hypoxia‐inducible factor (HIF) is involved in various biological processes such as erythropoiesis, anaerobic glycolytic metabolism, cell proliferation, and immunity (Yeo 2019). Erythropoietin (EPO) is a circulating glycoprotein hormone that promotes erythropoiesis by stimulating the proliferation of erythrocyte precursor cells and increases systemic oxygen‐carrying capacity to counteract tissue hypoxia (Scholz et al. 1990). In a hypoxic environment, HIF can enhance erythropoiesis by up‐regulating the expression of EPO, thus oxygen transport is increased (Haase 2013). Yak meat peptide significantly increased the mRNA expression level of EPO compared with yellow cattle peptide in a hypoxic environment (Figure 4). Additionally, it improved oxygen transport capacity by promoting erythropoiesis and increased hypoxia tolerance and antioxidant capacity in mice by reducing apoptosis and oxidative stress in myocardial tissue cells.

Under hypoxia, cells reduce the need for mitochondrial oxidative phosphorylation for energy supply and instead utilize anaerobic glycolysis to generate sufficient adenosine tri‐phosphate (ATP) to meet the energy needs of the organism. HIF increases the flux of anaerobic glycolysis by up‐regulating glucose transporter protein (GLUT‐1) and glycolytic enzyme transcription, which contributes to the body's production of ATP under hypoxic conditions (Kierans and Taylor 2021). CO produced by the catabolism of HO‐1 plays a role in regulating vascular tone and cell proliferation. Additionally, the catabolism of HO‐1 generates biliverdin. Biliverdin is then catalyzed by the enzyme cholangiase to form bilirubin that possesses antioxidant properties. It reduces the oxidative stress damage to cells caused by the reactive oxygen species generated by heme‐induced catabolism (Lee et al. 1997; Chen et al. 2019). Yak meat peptides significantly increased the mRNA expression levels of GLUT‐1 and HO‐1 compared to yellow cattle peptides (Figure 4). This resulted in improved anaerobic glycolysis of cells and oxygen transport in hypoxic mice, and reduced the damage caused by oxidative stress to cardiomyocytes.

HIF also induces the expression of vascular epidermal growth factor (VEGF), which promotes vascular neogenesis and increases oxygen transport in hypoxic tissues. Under hypoxic conditions, HIF upregulated VEGF gene expression to adapt to hypoxia, but yak meat peptide significantly reduced VEGF expression in myocardial tissues of hypoxic mice compared with yellow cattle peptide and casein (*p* < 0.05). It has been shown that higher VEGF expression leads to increased vascular permeability, which ultimately causes tissue edema inducing the acute plateau response. In contrast, low expression levels of VEGF can attenuate the damage to vascular endothelial function caused by contractile factors and eliminate the acute plateau response (Fan et al. 2020). Therefore, compared with yellow cattle peptide, yak meat peptide reduced the VEGF expression level in mouse myocardial tissues. This is likely to reduce the oxidative stress damage to the organism caused by hypoxia. It is further expressed in the process of slowly adapting to hypoxia, promoting neovascularization and enhancing the hypoxia tolerance of mice.

### 3.6 Effect of Yak Meat Peptide on Protein Expression of HIF, EPO, VEGFA, GLUT1, and HO‐1 in Mouse Myocardial Tissue

**FIGURE 5:**
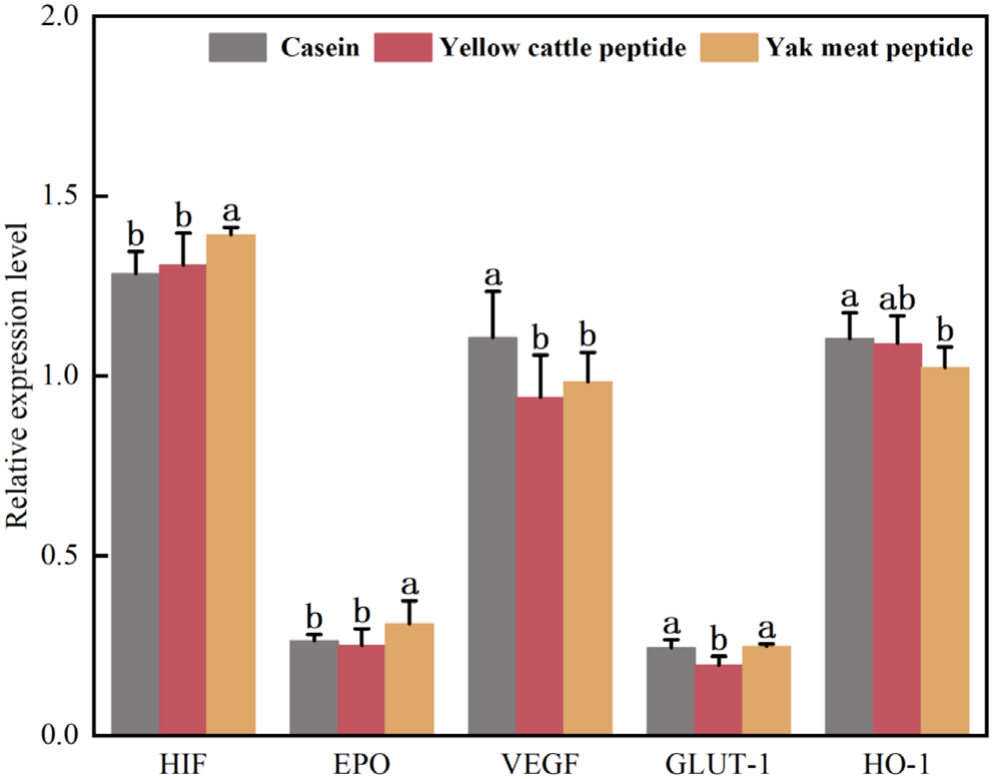
The effects of yak meat peptide on the expression of hypoxia‐related proteins in mouse myocardial tissue. HIF, EPO, VEGFA, GLUT‐1, and HO‐1 represent hypoxia‐inducible factors, erythropoietin, vascular epidermal growth factor, glucose transporter protein, and heme oxygenase in mouse myocardial tissues, respectively. Different lowercase letters for each gene indicate the differences among samples (*p* < 0.05).

As shown in Figure 5, the protein expression levels of VEGF and HO‐1 were significantly lower in the yak meat peptide compared to the casein (*p* < 0.05). The protein expression levels of HIF, EPO, and GLUT‐1 were significantly increased in the yak meat peptide group compared with the yellow cattle peptide group (*p* < 0.05). HIF mediates the transcription of the HO‐1 gene in response to hypoxia and regulates the organism, but the expression levels of HO‐1 protein in the yak meat peptide and the casein were exactly the opposite of those of mRNA. This is consistent with the findings of Jernigan et al. (2001). Although there is evidence that HO‐1 is functionally upregulated during hypoxia, it was not possible to detect elevated HO‐1 expression levels in protein immunoblots, which may be related to organ sensitization. It has been shown that heme, a key degradation by‐product of hemoglobin oxidation, may also affect the functional expression of HO‐1 in myocardial tissues when there is not enough heme available for HO‐1 to catalyze catabolism (Kassa et al. 2016). The increase in the expression of HIF protein strengthened the adaptation of the myocardial tissue cells to the hypoxic environment in mice, and increased the hypoxic tolerance in mice. The increase in the expression of EPO protein contributed to the erythropoietic production and increased the oxygen transport capacity. This also reduced apoptosis and oxidative stress in myocardial tissue cells, and improved hypoxia tolerance and antioxidant capacity in mice. Increased GLUT‐1 protein expression promoted glucose uptake and enhanced anaerobic glycolysis in cells, and improved hypoxia tolerance in mice.

## 4 Conclusion

In conclusion, yak meat peptides, which are generated through the digestion of yak meat protein, have a significant impact on enhancing immunity, antioxidant capacity, and hypoxia tolerance of mice. This is achieved by increasing the content of immune cells and improving oxygen transport. The increased immune cell content helps strengthen the body's defense system, enabling mice to better resist various pathogens and maintain overall health. Moreover, the enhanced oxygen transport ensures that cells receive sufficient oxygen supply, especially in hypoxic conditions. This is crucial for the survival and normal functioning of cells. The improved antioxidant capacity of yak meat peptides helps reduce oxidative stress damage caused by free radicals, protecting cells from damage and maintaining their normal physiological functions. Overall, these findings provide a solid scientific basis for the further development and utilization of yak meat. By exploring and harnessing the beneficial effects of yak meat peptides, we can potentially develop new functional foods or dietary supplements that can promote human health and well‐being.

## Author Contributions

**Gaiming Zhao:** funding acquisition (equal), methodology (equal), resources (equal), supervision (equal). **Ying Zhang:** software (equal), validation (equal), writing – original draft (equal), writing – review and editing (equal). **Ke Wang:** conceptualization (equal), data curation (equal), supervision (equal), visualization (equal). **Long Xu:** investigation (equal), methodology (equal), supervision (equal), validation (equal). **Mancai Yin:** formal analysis (equal), investigation (equal), validation (equal). **Xiangying Kong:** investigation (equal), supervision (equal), visualization (equal). **Chaozhi Zhu:** conceptualization (equal), formal analysis (equal), supervision (equal), writing – review and editing (equal).

## Conflicts of Interest

The authors declare no conflicts of interest.

## Supporting information


**Table S1.** Configuration of electrolyte stock solution.

**Table S2.** Primer sequence.

## Data Availability

The data will be made available on request.
