# Supplementary material for: Yak ( Bos grunniens ) Meat Peptides: Effects on Immunity, Hypoxia Tolerance, and Antioxidant Capacity in Hypotonic Hypoxia Mice
Source: Food Sci Nutr. 2025 Jun 27;13(7):e70522. doi: 10.1002/fsn3.70522 (PMC12203407; doi:10.1002/fsn3.70522)
Supplement: Supplementary file 1 — Table S1. Configuration of electrolyte stock solution. [file FSN3-13-e70522-s001.docx]

| Supplementary Table 1 Configuration of electrolyte stock solution | | | |
| --- | --- | --- | --- |
| Salt in solution | SSF(mmol/L)  (pH=7) | SGF(mmol/L)  (pH=3) | SUF(mmol/L)  (pH=7) |
| KCl | 15.1 | 6.9 | 6.8 |
| KH_2_PO_4_ | 3.7 | 0.9 | 0.8 |
| NaHCO_3_ | 13.6 | 25 | 85 |
| MgCl_2_(H_2_O)_6_ | 0.15 | 0.12 | 0.33 |
| (NH_4_)_2_CO_3_ | 0.06 | 0.5 | - |
| HCl | 1.1 | 15.6 | 8.4 |
| CaCl_2_(H_2_O_2_)_2_ | 1.5 | 0.15 | 0.6 |
| NaCl | - | 47.2 | 38.4 |

Note: SSF, SGF, and SUF stand for simulated saliva, simulated gastric fluid, and simulated intestinal fluid, respectively.
