# Supplementary material for: Yak ( Bos grunniens ) Meat Peptides: Effects on Immunity, Hypoxia Tolerance, and Antioxidant Capacity in Hypotonic Hypoxia Mice
Source: Food Sci Nutr. 2025 Jun 27;13(7):e70522. doi: 10.1002/fsn3.70522 (PMC12203407; doi:10.1002/fsn3.70522)
Supplement: Supplementary file 2 — Table S2. Primer sequence. [file FSN3-13-e70522-s002.docx]

| Supplementary Table 2 Primer sequence | |
| --- | --- |
| Primer name | Primer sequence |
| HIF-1α-R | CAAGTCAGCAACGTGGAAGG |
| HIF-1α-F | ATCAGCACCAAGCACGTCAT |
| EPO-F | GCTCTCAGAAGCCATCCTGC |
| EPO-R | GTGAGGCTACGAAGACCACTG |
| VEGF-R | CTGTGCAGGCTGCTGTAACG |
| VEGF-F | GCTCATTCTCTCTATGTGCTGGC |
| Glut1-R | TCAACACGGCCTTCACTG |
| Glut1-F | CACGATGCTCAGATAGGACATC |
| HO-1-R | GATGGCGTCACTTCGTCAGAG |
| HO-1-F | CCACTGGAGGAGCGGTGTC |
| β-actin-R | CTAAGGCCAACCGTGAAAAGAT |
| β-actin-F | GACCAGAGGCATACAGGGACA |
